# Peristaltic pumping of Boron Nitride-Ethylene Glycol nanofluid through a complex wavy micro-channel under the effect of induced magnetic field and double diffusive

**DOI:** 10.1038/s41598-023-29301-9

**Published:** 2023-02-14

**Authors:** Sameh A. Hussein, Nabil T. Eldabe

**Affiliations:** 1 Department of Mathematics and Computer Science, Faculty of Science, Zagazig University, Zagazig, Egypt; 2 Department of Mathematics, Faculty of Education, Ain Shams University, Cairo, Egypt

**Keywords:** Mathematics and computing, Nanoscience and technology

## Abstract

The main objective of this work is to present a comprehensive study that scrutinize the influence of DD convection and induced magnetic field on peristaltic pumping of Boron Nitride—Ethylene Glycol nanofluid flow through a vertical complex irregular microchannel. Experimental study showed that the nanofluid created by suspending Boron Nitride particles in a combination of Ethylene Glycol exhibited non-Newtonian characteristics. Further, the Carreau's fluid model provides accurate predictions about the rheological properties of BN-EG nanofluid. In order to imitate complicated peristaltic wave propagation conditions, sophisticated waveforms are forced at the walls. The essential properties of Brownian motion and thermophoresis phenomena are also included in simulating of heat equation as well as viscous dissipation. Mathematical simulation is performed by utilizing the lubrication approach. The resulting nonlinear coupled differential equation system is solved numerically using the built-in command (ND Solve function) in the Mathematica program. Numerical and pictorial evidence is used to illustrate the importance of various physiological features of flow quantities. The major findings demonstrated that the thermal resistance is observed to rise as the Soret and Dufour numbers increase, while the dissolvent concentration and nanoparticles volume fraction have the opposite effect.

## Introduction

Peristaltic flow has received a lot of attention in recent years due to its importance in industry and physiology. Peristalsis is involved in the transport of urine from the kidney to the bladder, the vasomotion of tiny blood arteries, the movement of chyme, and numerous reproductive processes. The peristaltic mechanism is often used in industry to run roller and finger pumps operate. The Newtonian constitutive equation can be used to describe the rheology of fluid materials driven by peristaltic mechanisms in a variety of flow scenarios. The rheology of urine, for instance, can be well characterized if it is assumed to be a Newtonian fluid. However, there are a variety of instances in which a Newtonian fluid is inappropriate. Many biological fluids particularly blood, chyme, and spermatic fluid, are some instances of non-Newtonian fluids. Certainly, Engineers, physicists, modelers, numerical analysts, and mathematicians face unique challenges when dealing with non-Newtonian fluid mechanics. Non-Newtonian fluid flows are important not just because of their technological importance, but also because of the fascinating mathematical aspects that their governing equations present. The rheological behavior of non-Newtonian fluids is extremely complex and finding a universal constitutive relation that applies to all non-Newtonian fluids is impossible. Many attempts have been made to examine peristaltic motion of non-Newtonian fluids theoretically since Latham’s^1^ first investigation on peristaltic motion of a Newtonian fluid. Hayat et al.^2^, Hayat and Ali^3^, Wang et al.^4^, Ali et al.^5^, Srinivas and Kothandapani^6^, Tripathi et al.^7^, and Abbasi et al.^8^ have all conducted noteworthy investigations on these flows.

Due to their abundance in both ecology and technology, non-Newtonian fluids are found in many different domains. Such liquids have been discovered to be advantageous in Melts of polymer, detergents in liquid form, Coatings and multi grades of oils, among other things. Carreau fluid, one of the models proposed for non-Newtonian fluids, is considered extremely favorable in predicting the wide and varied properties of fluids. It is worth noting that the addendum of nanoparticles to liquid alters both the mechanical and thermal characteristics of the liquid, and very little dedication has been dedicated to the study of the rheological characteristics of nanofluids. Hence, the non-Newtonian behavior and attributes of nanofluid is especially focused here in order to predict its rheological properties. Several considerable efforts have been made to analyze the non-Newtonian fluids' flow for industrial applications, as evidenced by references^9–15^. According to Zhi et al.^16^, depicted that by utilizing BN nanoparticles, water's thermal conductivity could be enhanced by up to 2.6 times at a fraction of 6 vol%.

Heat transfer process improvement is a major issue in both industrial and biological sciences. The heat transfer between tissues is caused by blood circulation in the body. As a result, the effect of heat on tissues and the mechanism of heat transfer between tissues has been extensively studied. The cooling system of automobile engines and nuclear reactors is critical to their efficiency as heat radiators are used to prevent engines from overheating due to friction. By transferring heat away from the heat source ethylene glycol, water, and engine oil, among other common fluids, are used to cool various industrial appliances. Nanofluid is a homogeneous combination of nanoparticles and fluids^17^. The thermophysical performance of the rheological features of mono-nanofluids, compact and hybrid heat exchangers containing $Al2O3$ and $CuO$ nanoparticles at low concentration was studied by Asoken et al.^18^. They found that when compared to $CuO$, the thermal conductivity of $Al2O3-CuO$ increased by 2.3%, and by 3.6% percent when compared to $Al2O3$. Jiang et al.^19^ investigated the effect of nanoparticle shape factor on nanofluid in a mini channel with combined forced and thermocapillary convective. Blade-shaped nanomaterial has a higher thermal efficiency than spherical nanomaterial. The VVER-1000 nuclear reactor duty operating cycle augmentation and safety margin were verified using a water/silver nanofluid by Sadatee et al.^20^. Buongiorno^21^ studied convective transport in nanofluids using the seven slip processes to explain the thermophysical relationship between relative fluid and nanomaterial velocity. Several attempts to describe the use of nanofluid could be identified in the literature^22–49^.

Diffusion effects are not taken into account in the literature that has been studied so far. However, because they are used throughout numerous industrial and engineering fields, non-Newtonian liquids exhibiting diffusion effects have attracted the interest of researchers. The process of diffusion happens in a flowing fluid as a result of changes in the temperature and concentration gradients that are responsible for producing flux in a liquid stream. The thermo-diffusion phenomena are caused by the existence of concentration fluxes induced by thermal changes, diffusion-thermo effects are produced by the energy created by the mass fluxes of the concerned particles^50^ that are investigated the monoatomic gases thermo-diffusion coefficient parameter. Whereas^51^ discusses the relationship between important parameters that influence the transport of heat and mass. Temperature gradients during the processes of mass and heat transport not only result in energy gradients but also get change in the temperature^52^. Illustrates the computational effects of DD-convection on a stretched sheet. In^53–58^, some pertinent double diffusion studies for peristaltic movement is given.

It is discovered from the examined literature that no single investigate has been demonstrated in order to examine peristaltic transportation of Boron Nitride—Ethylene Glycol nanofluid flow through a vertical complex irregular microchannel with combined effectiveness of thermo diffusion and diffusion-thermo impacts by taking induced magnetic field into account. The suggested research project is therefore provided to fill these issues. Motivated by the importance of mixed convection with magnetic nanomaterials in peristaltic flow in variety of applications (as evidenced by the above-aforementioned literature), the author's main goal is to present a study that investigates the influence of double diffusion convection and induced magnetic field on the peristaltic flow of BN-EG nanofluid through a vertical complex irregular channel. The current work is pertinent to the peristaltic propulsion mechanism-based electromagnetic biomaterials micro—scale pumps that simulate actual working fluids^59^. These pumps have a huge promise in bio-inspired transportable intravenous dripping systems for medical procedures in the twenty-first century since they prevent contamination issues, reduce maintenance requirements, and also achieve improved longevity and competence. The current paper is divided into different sections, with the following information’s: Sect. “Basic equations” identifies the fundamental governing equations; Sect. “Mathematical formulation” provides mathematical perspectives of the non-Newtonian fluid model for 2-dimensional flows with movement patterns of nanofluids through DD convection aspects; Sect. “Discussion and graphical illustrations” serves to highlight the proposed framework and illustrates the graphical reasonable interpretation of obtained results; and Sect. “Conclusions” describes the problem’s concluding remarks.

## Basic equations

(i) Maxwell’s equation

1 $$\nabla .\overline{{\varvec{E}} }=0,\nabla .\overline{{\varvec{H}} }=0,$$

2 $$\nabla \times \overline{{\varvec{H}} }=\overline{{\varvec{J}} }, \overline{{\varvec{J}} }=\sigma \left\{\overline{{\varvec{E}} }+{\mu }_{e}\left({\varvec{V}}\times \overline{{\varvec{H}} }\right)\right\},$$

3 $$\nabla \times \overline{{\varvec{E}} }=-{\mu }_{e}\frac{\partial \overline{{\varvec{H}}}}{\partial t },$$

The induction equation can now be calculated by applying Eqs. (1) through (3) as follows:

4 $$\frac{\partial {\overline{{\varvec{H}}} }^{+}}{\partial t}=\nabla \times \left({\varvec{V}}\times {\overline{{\varvec{H}}} }^{+}\right)+\frac{1}{\chi }{\nabla }^{2}{\overline{{\varvec{H}}} }^{+},$$

where ${\mu }_{e}$ is the magnetic permeability symbol, $\chi ={\sigma \mu }_{e}$ represents the diffusivity of magnetization, g symbolizes acceleration, $\sigma$ indicates electrical conductivity, $t$ the time, ${\varvec{E}}$ denotes the induced electric field, ${\varvec{J}}$ symbolizes current density, and ${\varvec{V}}$ represents the vector of velocity.

(ii) Continuity equation

5 $$div {\varvec{V}}=0.$$

(iii) Navier-Stokes equation

6 $${\rho }_{f}\left(\frac{d{\varvec{V}}}{dt}\right)=div {\varvec{\tau}}-{\mu }_{e}\left({\overline{{\varvec{H}}} }^{+}.\nabla \right){\overline{{\varvec{H}}} }^{+}-\nabla \left(\frac{1}{2}{\mu }_{e}{\left({\overline{{\varvec{H}}} }^{+}\right)}^{2}\right)+{{\varvec{F}}}_{g},$$

where ${{\varvec{F}}}_{g}$ body force, which is defined as:

7 $${{\varvec{F}}}_{g}=g\left\{\left(1-{\Theta }_{0}\right){\rho }_{f0}\left\{{\beta }_{T}\left(T-{T}_{0}\right)+{\beta }_{C}\left(C-{C}_{0}\right)\right\}-\left({\rho }_{p}-{\rho }_{f0}\right)\left(\Theta -{\Theta }_{0}\right)\right\},$$

where ${\beta }_{T}$ denotes coefficient of volumetric heat expansion., ${\beta }_{C}$ demonstrates the coefficient of volumetric solute expansion, $C$ stands for concentration, ${\rho }_{p}$ indicates density of nanoparticles, ${\rho }_{f0}$ represents fluid density at ${T}_{0}$, ${\rho }_{f}$ symbolizes fluid density, $\Theta$ is nanoparticle volume fraction, $T$ indicates the temperature of the fluid, $\left(\frac{d}{dt}\right)$ denotes the material derivative with respect to the time.

(iv) Equations for the volume fractions of nanoparticles, the concentration of solute, and heat energy

The heat energy, concentration of solute, and nanoparticle volume fraction^53–58^: are calculated utilizing Oberbeck-Boussinesq hypothesis as follows:

8 $${\left(\rho c\right)}_{f}\left(\frac{d{\varvec{T}}}{dt}\right)=k{ \nabla }^{2}T+{\left(\rho c\right)}_{p}\left\{{D}_{B}\left(\nabla \Theta .\nabla T\right)+\frac{{D}_{T}}{{T}_{0}}\left(\nabla T.\nabla T\right)\right\}+{D}_{TC}{ \nabla }^{2}C,$$

9 $$\frac{d{\varvec{C}}}{dt}={D}_{s}{ \nabla }^{2}C+{D}_{CT}{ \nabla }^{2}T,$$

10 $$\frac{d\boldsymbol{\Theta }}{dt}={D}_{B}{ \nabla }^{2}\Theta +\left(\frac{{D}_{T}}{{T}_{0}}\right){ \nabla }^{2}T,$$

where ${D}_{B}$ stands for the coefficient of Brownian diffusion, ${D}_{TC}$ indicates Dufour diffusion, ${D}_{T}$ indicates the coefficient of thermophoretic diffusion, ${D}_{CT}$ represents Soret diffusively, ${D}_{s}$ symbolizes the diffusively of solute, ${\left(\rho c\right)}_{f}$ stands for fluid heat capacity, $k$ denotes thermal conductivity, and ${\left(\rho c\right)}_{p}$ is effective nanoparticle heat capacity.

## Mathematical formulation

**Figure 1:**
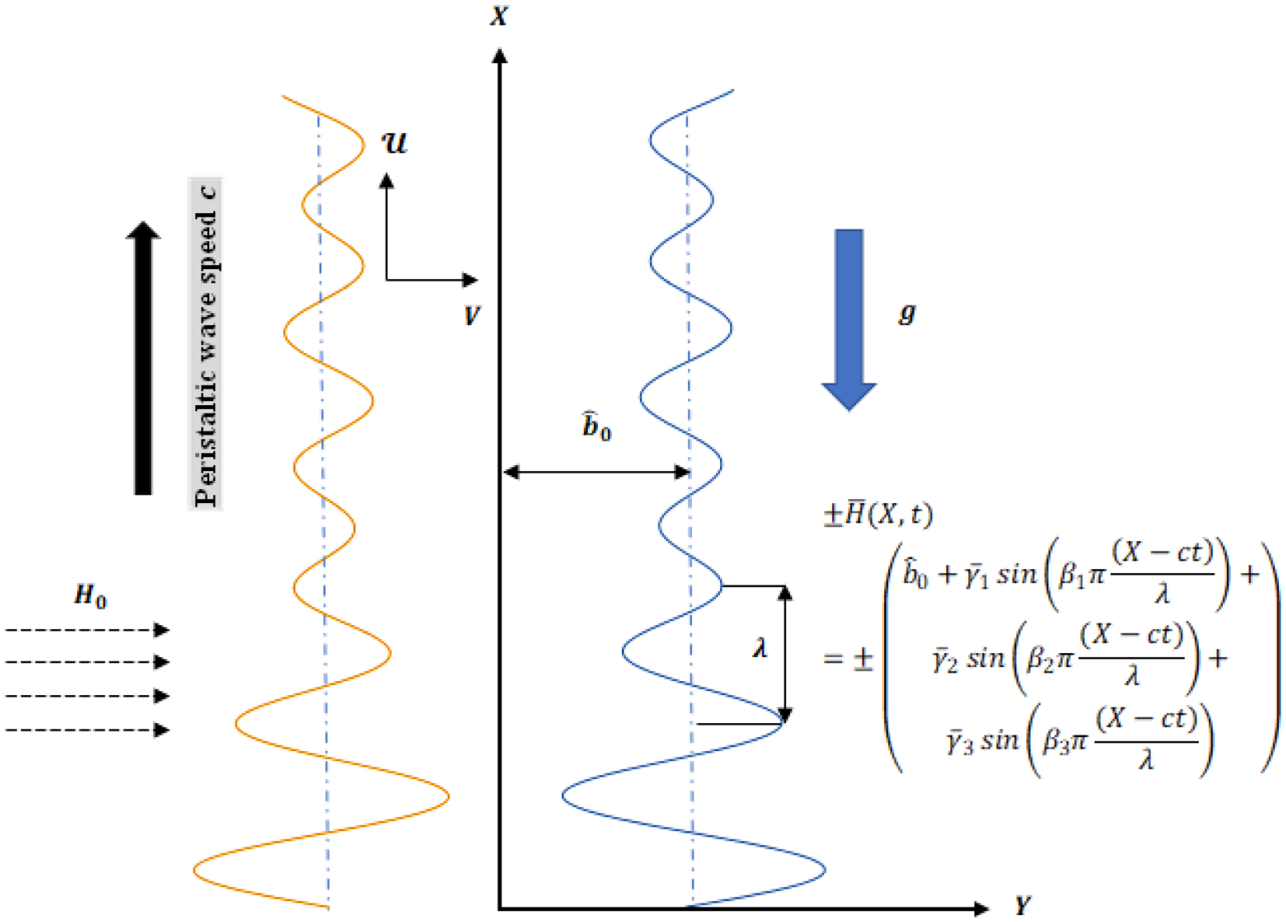
Flow configuration.

Consider a Boron Nitride nanoparticles in suspension Ethylene Glycol nanofluid that is electrically conducting and flowing through a complex wavy two-dimensional microchannel in the presence of DD convection and induced magnetic field. The fluid is initially at rest, and the peristaltic transport is produced by sinusoidal wave constructions. The direction of wave motion is sustained along the $X$-axis, while the $Y$-axis is orthogonal to it. The induced magnetic field $H({h}_{X}(X,Y, t),{ H}_{0}+{ h}_{Y}(X,Y, t), 0)$ and all magnetic fields combined ${H}^{+}({h}_{X}(X,Y, t),{ H}_{0}+{ h}_{Y}(X,Y, t), 0)$ are created by a perpendicular, intense magnetic field that is sustained. The following competent mathematical relations are utilized to define the equations of sinusoidal walls for the geometry of flow with limited length as portrayed in Fig. 1 (see^60,61^):

11 $$\begin{array}{ll}\pm \overline{H }\left(X,t\right)=\pm \left(\begin{array}{c}{\widehat{b}}_{0}+{\overline{\gamma }}_{1}\mathit{sin}\left({\beta }_{1}\pi \frac{\left(X-ct\right)}{\lambda }\right)+\\ {\overline{\gamma }}_{2}\mathit{sin}\left({\beta }_{2}\pi \frac{\left(X-ct\right)}{\lambda }\right)+\\ {\overline{\gamma }}_{3}\mathit{sin}\left({\beta }_{3}\pi \frac{\left(X-ct\right)}{\lambda }\right)\end{array}\right),& \\ & \end{array}$$

Here$, \overline{H },-\overline{H } , \lambda , t, {\widehat{b}}_{0}, c, X , {\overline{\gamma }}_{j} ( j = 1-3)$ ψ and $L$ are symbolizes both the upper and lower walls, wavelength, time, half the channel's width, Wave speeds, axial coordinates, and complex wave amplitudes such that ${\widehat{b}}_{0} \le \sum_{j=1}^{3}{\overline{\gamma }}_{j}$ and flow regime’s length, correspondingly.

The fluid velocity in two-dimensional can be written as:

12 $$V=(\mathcal{U}(X,Y, t),V(X,Y, t),0),$$

Using Eq. (12), the Eqs. (4)– (10) in laboratory frame $(X,Y)$ become^53–58^:

13 $$\frac{\partial \mathcal{U}}{\partial X}+\frac{\partial V}{\partial Y}=0,$$

14 $$ \begin{aligned}  & {\rho }_{f}\left(\frac{\partial \mathcal{U}}{\partial t}+\mathcal{U}\frac{\partial \mathcal{U}}{\partial X}+V\frac{\partial \mathcal{U}}{\partial Y}\right)=-\frac{\partial P}{\partial X}+\left(\frac{\partial {S}_{XX}}{\partial X}+\frac{\partial {S}_{XY}}{\partial Y}\right)\\ &\quad-{\mu }_{e}\left(\frac{\partial {{H}^{+}}^{2}}{\partial Y}\right)+{\mu }_{e}\left({h}_{X}\frac{\partial {h}_{X}}{\partial X}+{h}_{Y}\frac{\partial {h}_{X}}{\partial Y}+{H}_{0}\frac{\partial {h}_{X}}{\partial Y}\right) \\ &\quad+ g\left\{\left(1-{\Theta }_{0}\right){\rho }_{f0}\left\{{\beta }_{T}\left(T-{T}_{0}\right)+{\beta }_{C}\left(C-{C}_{0}\right)\right\}-\left({\rho }_{p}-{\rho }_{f0}\right)\left(\Theta -{\Theta }_{0}\right)\right\},\end{aligned} $$

15 $${\rho }_{f}\left(\frac{\partial V}{\partial t}+\mathcal{U}\frac{\partial V}{\partial X}+V\frac{\partial V}{\partial Y}\right)=-\frac{\partial P}{\partial Y}+\left(\frac{\partial {S}_{YX}}{\partial X}+\frac{\partial {S}_{YY}}{\partial Y}\right)-{\mu }_{e}\left(\frac{\partial {{H}^{+}}^{2}}{\partial Y}\right)+{\mu }_{e}\left({h}_{X}\frac{\partial {h}_{Y}}{\partial X}+{h}_{Y}\frac{\partial {h}_{Y}}{\partial Y}+{H}_{0}\frac{\partial {h}_{Y}}{\partial Y}\right),$$

16 $$ \begin{aligned}&{\left(\rho C\right)}_{f}\left(\frac{\partial T}{\partial t}+\mathcal{U}\frac{\partial T}{\partial X}+V\frac{\partial T}{\partial Y}\right)=k\left(\frac{{\partial }^{2}T}{\partial {X}^{2}}+\frac{{\partial }^{2}T}{\partial {Y}^{2}}\right)\\ &\quad{+\;\left(\rho C\right)}_{p}\left\{{D}_{B}\left(\frac{\partial T}{\partial X}\frac{\partial \Theta }{\partial X}+\frac{\partial T}{\partial Y}\frac{\partial \Theta }{\partial Y}\right)+\frac{{D}_{T}}{{T}_{0}}\left[{\left(\frac{\partial T}{\partial X}\right)}^{2}+{\left(\frac{\partial T}{\partial Y}\right)}^{2}\right]\right\}\\ &\quad+{\varvec{S}}.{\varvec{L}}+{D}_{TC}\left(\frac{{\partial }^{2}C}{\partial {X}^{2}}+\frac{{\partial }^{2}C}{\partial {Y}^{2}}\right),\end{aligned} $$

17 $$\left(\frac{\partial C}{\partial t}+\mathcal{U}\frac{\partial C}{\partial X}+V\frac{\partial C}{\partial Y}\right)={D}_{s}\left(\frac{{\partial }^{2}C}{\partial {X}^{2}}+\frac{{\partial }^{2}C}{\partial {Y}^{2}}\right)+{D}_{CT}\left(\frac{{\partial }^{2}T}{\partial {X}^{2}}+\frac{{\partial }^{2}T}{\partial {Y}^{2}}\right),$$

18 $$\left(\frac{\partial\Theta }{\partial t}+\mathcal{U}\frac{\partial\Theta }{\partial X}+V\frac{\partial\Theta }{\partial Y}\right)={D}_{B}\left(\frac{{\partial }^{2}\Theta }{\partial {X}^{2}}+\frac{{\partial }^{2}\Theta }{\partial {Y}^{2}}\right)+\frac{{D}_{T}}{{T}_{0}}\left(\frac{{\partial }^{2}T}{\partial {X}^{2}}+\frac{{\partial }^{2}T}{\partial {Y}^{2}}\right).$$

The experimental study established by Zyla et al.^13^ revealed that the BN-EG nanofluid exhibited non-Newtonian behavior. In this analysis, the author hypothesized that the Carreau's fluid model constitutes excellent predictions about the rheological properties of BN-EG nanofluid. The additional stress tensor for Carreau's model is therefore given as:

19 $$S={A}_{1}\eta .$$

where, ${A}_{1}$ is a symbol for the “first Rivlin-Erickson tensor” and $\eta$ stands for the perceived viscosity is described as^9–16^:

$$\eta =\left[{\eta }_{\infty }+\frac{\left({\eta }_{0}-{\eta }_{\infty }\right)}{{\left[1+{\left(\alpha \dot{\gamma }\right)}^{2}\right]}^\frac{n}{2}}\right],$$

20 $$\dot{\gamma }=\sqrt{2 tr {D}^{2}},D=\frac{1}{2}{A}_{1}and {A}_{1}=grad V+{\left(grad v\right)}^{T},$$

Here ${\eta }_{0}$ and ${\eta }_{\infty }$ denotes the viscosities with zero and infinite shear rates, respectively. Furthermore, $grad V ,\alpha , \dot{\gamma }$ and $n$ indicates the gradient of velocity, the fluid material parameter, the shear rate and index of the non-dimensional power law. Furthermore, $\overline{{\varvec{S}} }.\boldsymbol{ }{\varvec{L}}$ is the viscous heating where ${\varvec{L}}$ denotes the gradient of velocity.

The stress tensor with the viscosity parameter’s $\left(\beta =\frac{{\eta }_{\infty }}{{\eta }_{0}}\right)$ component form is computed as follows:

21 $${S}_{XX}=2{\eta }_{0}\left[1+\frac{{\alpha }^{2}n}{2}(\beta -1)\left\{2{\left(\frac{\partial \mathcal{U}}{\partial X}\right)}^{2}+2{\left(\frac{\partial V}{\partial Y}\right)}^{2}+{\left(\frac{\partial \mathcal{U}}{\partial Y}+\frac{\partial V}{\partial X}\right)}^{2}\right\}\right]\left[\frac{\partial \mathcal{U}}{\partial X}\right],$$

22 $${S}_{XY}={\eta }_{0}\left[1+\frac{{\alpha }^{2}n}{2}(\beta -1)\left\{2{\left(\frac{\partial \mathcal{U}}{\partial X}\right)}^{2}+2{\left(\frac{\partial V}{\partial Y}\right)}^{2}+{\left(\frac{\partial \mathcal{U}}{\partial Y}+\frac{\partial V}{\partial X}\right)}^{2}\right\}\right]\left[\frac{\partial \mathcal{U}}{\partial Y}+\frac{\partial V}{\partial X}\right],$$

23 $${S}_{YY}=2{\eta }_{0}\left[1+\frac{{\alpha }^{2}n}{2}(\beta -1)\left\{2{\left(\frac{\partial \mathcal{U}}{\partial X}\right)}^{2}+2{\left(\frac{\partial V}{\partial Y}\right)}^{2}+{\left(\frac{\partial \mathcal{U}}{\partial Y}+\frac{\partial V}{\partial X}\right)}^{2}\right\}\right]\left[\frac{\partial V}{\partial Y}\right].$$

Now utilizing fixed-frame Galilean transformations $(X,Y)$ and frame of the wave $\left(x,y\right)$ as:

$$x=X-ct,y=Y,$$

24 $$v\left(x,y\right)=V\left(X,Y, t\right),u\left(x,y\right)=\mathcal{U}\left(X,Y, t\right)-c,p\left(x,y\right)=P\left(X,Y, t\right),$$

as well as creating non-dimensional parameters as follows:

25 $$ \begin{aligned}  &  \mathcal{Y}=\frac{y}{{\widehat{b}}_{0}},\mathcal{X}=\frac{x}{\lambda } ,V=\frac{v}{c},\mathcal{U}=\frac{u}{c},\delta =\frac{{\widehat{b}}_{0}}{\lambda },t=\frac{ct}{\lambda },Re=\frac{{{\rho }_{f}c\widehat{b}}_{0}}{{\eta }_{0}},h=\frac{\overline{H}}{{\widehat{b} }_{0}},\\ & \theta =\frac{T-{T}_{0}}{{T}_{1}-{T}_{0}},\gamma =\frac{C-{C}_{0}}{{C}_{1}-{C}_{0}},\Omega =\frac{\Theta -{\Theta }_{0}}{{\Theta }_{1}-{\Theta }_{0}},u=\frac{\partial \Psi }{\partial y}, v=-\delta \frac{\partial \Psi }{\partial x},{h}_{X}=\frac{\partial \Phi }{\partial y},\\ & {h}_{y}=-\delta \frac{\partial \Phi }{\partial x},{R}_{m}=\sigma {\mu }_{e}{\widehat{b}}_{0}c,{S}_{1}=\frac{{H}_{0}}{c}\sqrt{\frac{{\mu }_{e}}{{\rho }_{f}}},E=-\frac{E}{{\mu }_{e}{H}_{0}c}, Pr=\frac{{\left(\rho C\right)}_{f}\nu }{k},Ln=\frac{\nu }{{D}_{B}},\\&{N}_{CT}=\frac{{D}_{CT}\left({T}_{1}-{T}_{0}\right)}{\left({C}_{1}-{C}_{0}\right){D}_{S}},{N}_{TC}=\frac{{D}_{TC}\left({C}_{1}-{C}_{0}\right)}{\left({T}_{1}-{T}_{0}\right)K}, P=\frac{{{\widehat{b}}_{0}}^{2}P\left(x\right)}{{\eta }_{0}c\lambda },{G}_{rT}=\frac{g{{\widehat{b}}_{0}}^{2}\left(1-{\Theta }_{0}\right){\rho }_{f0}{\beta }_{T}\left({T}_{1}-{T}_{0}\right)}{{\eta }_{0}c},\\& Br=PrEc,Ec=\frac{{c}^{2}}{{C}_{f}\left({T}_{1}-{T}_{0}\right)},{M=R}_{m} Re {S}_{1}^{2},{G}_{rc}=\frac{g{{\widehat{b}}_{0}}^{2}\left(1-{\Theta }_{0}\right){\rho }_{f0}{\beta }_{C}\left({C}_{1}-{C}_{0}\right)}{{\eta }_{0}c},\\& {G}_{rF}=\frac{g{{\widehat{b}}_{0}}^{2}\left({\rho }_{p}-{\rho }_{f0}\right)\left({\Theta }_{1}-{\Theta }_{0}\right)}{{\eta }_{0}c},{\gamma }_{1}=\frac{{\overline{\gamma }}_{1}}{{\widehat{b}}_{0}},{\gamma }_{2}=\frac{{\overline{\gamma }}_{2}}{{\widehat{b}}_{0}},{\gamma }_{3}=\frac{{\overline{\gamma }}_{3}}{{\widehat{b}}_{0}}, S=\frac{{\widehat{b}}_{0}}{{\eta }_{0}c}S,Le=\frac{\nu }{{D}_{s}}, \\& Nb=\frac{{\left(\rho C\right)}_{p}{D}_{B}\left({\Theta }_{1}-{\Theta }_{0}\right)}{k},Nt=\frac{{\left(\rho C\right)}_{p}{D}_{T}\left({T}_{1}-{T}_{0}\right)}{{T}_{0}k},We=\frac{\alpha c}{{\widehat{b}}_{0}},\beta =\frac{{\eta }_{\infty }}{{\eta }_{0}}.\end{aligned} $$

Here ${G}_{rF}$ denotes nanoparticle Grashof number, ${G}_{rT}$ stands for thermal expansion Grashof number, ${G}_{rc}$ indicates the Grashof number of solute, $\theta$ stands for dimensionless temperature, $\Omega$ is nanoparticle volume fraction distribution, $\gamma$ stands for the concentration of solute, $Nb$ denotes Brownian motion, $Le$ symbolizes Lewis number, $Nt$ indicates the parameter thermophoresis motion, $Pr$ symbolizes Prandtl number, $Ln$ denotes the Lewis number of nanoparticle, ${N}_{TC}$ indicates Dufour number, ${N}_{CT}$ symbolizes Soret number, $\delta$ stands for the numbers of the wave, ${R}_{m}$ represents magnetic Reynolds number, ${S}_{1}$ symbolizes Strommer’s number and $Re$ indicates Reynolds number.

The Eq. (11) in dimensionless form becomes:

26 $$h\left(x\right)=1+{\gamma }_{1}\mathit{sin}\left({\beta }_{1}\pi x\right)+{\gamma }_{2}\mathit{sin}\left({\beta }_{2}\pi x\right)+{\gamma }_{3}\mathit{sin}\left({\beta }_{2}\pi x\right).$$

Equations (22) and (23) naturally satisfy Eq. (13), and in the wave frame, Eqs. (14)–(21) becoming as follows:

27 $$Re \delta \left({\Psi }_{xy}{\Psi }_{y}-{\Psi }_{yy}{\Psi }_{x}\right)=-\frac{\partial p}{\partial x}+\left(\delta \frac{\partial {S}_{xx}}{\partial x}+\frac{\partial {S}_{xy}}{\partial y}\right)+Re {S}_{1}^{2}{\Phi }_{yy}+Re {S}_{1}^{2}\delta \left({\Phi }_{y}{\Phi }_{xy}-{\Phi }_{x}{\Phi }_{yy}\right)+{G}_{rT}\theta +{G}_{rC}\gamma -{G}_{rF}\Omega ,$$

28 $$Re {\delta }^{3}\left({\Psi }_{xy}{\Psi }_{x}-{\Psi }_{xx}{\Psi }_{y}\right)=-\frac{\partial p}{\partial y}+\left({\delta }^{2}\frac{\partial {S}_{xy}}{\partial x}+\delta \frac{\partial {S}_{yy}}{\partial y}\right)-Re {S}_{1}^{2}{ {\delta }^{2}\Phi }_{xy}-Re {S}_{1}^{2}{\delta }^{3}\left({\Phi }_{y}{\Phi }_{xx}-{\Phi }_{x}{\Phi }_{xy}\right),$$

29 $$Re Pr \delta \left({\Psi }_{y}{\theta }_{x}-{\Psi }_{x}{\theta }_{y}\right)=\left({\theta }_{yy}+{\delta }^{2}{\theta }_{xx}\right)+{N}_{TC}\left({\gamma }_{yy}+{\delta }^{2}{\gamma }_{xx}\right)+Nb\left({\delta }^{2}{\theta }_{x}{\Omega }_{x}+{\theta }_{y}{\Omega }_{y}\right)+Nt\left({\delta }^{2}{\left({\theta }_{x}\right)}^{2}+{\left({\theta }_{y}\right)}^{2}\right)+Br \left[{\Psi }_{yy}{S}_{xy}-{\delta }^{2}{\Psi }_{xx}{S}_{yx}\right],$$

30 $$Re Le \delta \left({\Psi }_{y}{\gamma }_{x}-{\Psi }_{x}{\gamma }_{y}\right)=\left({\gamma }_{yy}+{\delta }^{2}{\gamma }_{xx}\right)+{N}_{CT}\left({\theta }_{yy}+{\delta }^{2}{\theta }_{xx}\right),$$

31 $$Re Ln \delta \left({\Psi }_{y}{\Omega }_{x}-{\Psi }_{x}{\Omega }_{y}\right)=\left({\Omega }_{yy}+{\delta }^{2}{\Omega }_{xx}\right)+\frac{Nt}{Nb}\left({\theta }_{yy}+{\delta }^{2}{\theta }_{xx}\right),$$

32 $${\Psi }_{y}-\delta \left(\frac{\partial \Psi }{\partial y}\frac{\partial\Phi }{\partial x}-\frac{\partial \Psi }{\partial x}\frac{\partial\Phi }{\partial y}\right)+\frac{1}{{R}_{m}}\left(\frac{{\partial }^{2}\Phi }{\partial {y}^{2}}+{\delta }^{2}\frac{{\partial }^{2}\Phi }{\partial {x}^{2}}\right)=E.$$

Now, by implementing the lubrication approach $(\delta << 1)$ with low, but limited Reynolds number Eqs. (26)–(31) have now been simplified as:

33 $$0=-\frac{\partial p}{\partial x}+\left(\frac{\partial {S}_{xy}}{\partial y}\right)+Re {S}_{1}^{2}\frac{{\partial }^{2}\Phi }{\partial {y}^{2}}+{G}_{rT}\theta +{G}_{rC}\gamma -{G}_{rF}\Omega ,$$

34 $$0=-\frac{\partial p}{\partial y},$$

35 $$\frac{{\partial }^{2}\theta }{\partial {y}^{2}}+{N}_{TC}\frac{{\partial }^{2}\gamma }{\partial {y}^{2}}+Nb\left(\frac{\partial\Omega }{\partial y}\frac{\partial \theta }{\partial y}\right)+Nt{\left(\frac{\partial \theta }{\partial y}\right)}^{2}+Br{\varvec{\Phi}}=0,$$

36 $$\frac{{\partial }^{2}\gamma }{\partial {y}^{2}}+{N}_{CT}\frac{{\partial }^{2}\theta }{\partial {y}^{2}}=0,$$

37 $$\frac{{\partial }^{2}\Omega }{\partial {y}^{2}}+\frac{Nt}{Nb}\left(\frac{{\partial }^{2}\theta }{\partial {y}^{2}}\right)=0,$$

38 $$\frac{{\partial }^{2}\Phi }{\partial {y}^{2}}={R}_{m}\left(E-\frac{\partial \Psi }{\partial y}\right).$$

39 $${S}_{xy}=\frac{{\partial }^{2}\Psi }{\partial {y}^{2}}\left[1+\frac{n}{2}(\beta -1){We}^{2}{\left(\frac{{\partial }^{2}\Psi }{\partial {y}^{2}}\right)}^{2}\right].$$

40 $${\varvec{\Phi}}=\left[1+\frac{n}{2}(\beta -1){We}^{2}{\left(\frac{{\partial }^{2}\Psi }{\partial {y}^{2}}\right)}^{2}\right]{\left(\frac{{\partial }^{2}\Psi }{\partial {y}^{2}}\right)}^{2}.$$

As a consequence of utilizing Eq. (39) and omitting pressure from Eqs. (33) and (34), we obtain the following utterance:

41 $$\frac{{\partial }^{2}}{\partial {y}^{2}}\left[\frac{{\partial }^{2}\Psi }{\partial {y}^{2}}\left[1+\frac{n}{2}(\beta -1){We}^{2}{\left(\frac{{\partial }^{2}\Psi }{\partial {y}^{2}}\right)}^{2}\right]\right]+Re {S}_{1}^{2}\frac{{\partial }^{3}\Phi }{\partial {y}^{3}}+{G}_{rT}\frac{\partial \theta }{\partial y}+{G}_{rC}\frac{\partial \gamma }{\partial y}-{G}_{rF}\frac{\partial \Omega }{\partial y}=0.$$

Also, by substitution with the Eq. (38) in Eq. (41) we can obtain the following utterance:

42 $$\frac{{\partial }^{2}}{\partial {y}^{2}}\left[\frac{{\partial }^{2}\Psi }{\partial {y}^{2}}\left[1+\frac{n}{2}(\beta -1){We}^{2}{\left(\frac{{\partial }^{2}\Psi }{\partial {y}^{2}}\right)}^{2}\right]\right]-{R}_{m} Re {S}_{1}^{2}\frac{{\partial }^{2}\Psi }{\partial {y}^{2}}+{G}_{rT}\frac{\partial \theta }{\partial y}+{G}_{rC}\frac{\partial \gamma }{\partial y}-{G}_{rF}\frac{\partial \Omega }{\partial y}=0.$$

Then,

43 $$\frac{{\partial }^{2}}{\partial {y}^{2}}\left[\frac{{\partial }^{2}\Psi }{\partial {y}^{2}}\left[1+\frac{n}{2}(\beta -1){We}^{2}{\left(\frac{{\partial }^{2}\Psi }{\partial {y}^{2}}\right)}^{2}\right]\right]-{M}^{2}\frac{{\partial }^{2}\Psi }{\partial {y}^{2}}+{G}_{rT}\frac{\partial \theta }{\partial y}+{G}_{rC}\frac{\partial \gamma }{\partial y}-{G}_{rF}\frac{\partial \Omega }{\partial y}=0.$$

Following is a description of the boundary restrictions for the given problem in wave frame:

44 $$\Psi =0,\frac{{\partial }^{2}\Psi }{\partial {y}^{2}}=0 at y=0,$$

45 $$\Psi =F,\frac{\partial \Psi }{\partial y}=-1 at y=h\left(x\right),$$

46 $$\frac{\partial\Phi }{\partial y}=0 at y=0,$$

47 $$\Phi =0, \frac{{\partial }^{2}\Phi }{\partial {y}^{2}}={R}_{m}\left(E+1\right) at y=h\left(x\right),$$

48 $$\theta =0, at y=0 and \theta =1 at y=h\left(x\right),$$

49 $$\frac{\partial\Omega }{\partial y}=0, at y=0 and\Omega =1 at y=h\left(x\right),$$

50 $$\frac{\partial \gamma }{\partial y}=0, at y=0 and \gamma =1 at y=h\left(x\right).$$

where, $F$ denotes the rate of wave frame time mean flow, which is related to time mean flow $(Q)$ by the equations $Q=F+1$ and $F={\int }_{0}^{h\left(x\right)}\frac{\partial \Psi }{\partial y} dy$.

The pressure rise $\Delta p$, and heat transfer rate $Z$ in non- dimensional form are defined as:

51 $${\mathrm{\Delta p}}_{\uplambda }={\int }_{0}^{1}\frac{dp}{dx} dx,$$

52 $$Z=\frac{\partial \left(h\left(x\right)\right)}{\partial x}{\left.\frac{\partial \theta }{\partial y}\right|}_{y=h\left(x\right)}.$$

## Discussion and graphical illustrations

**Figure 2:**
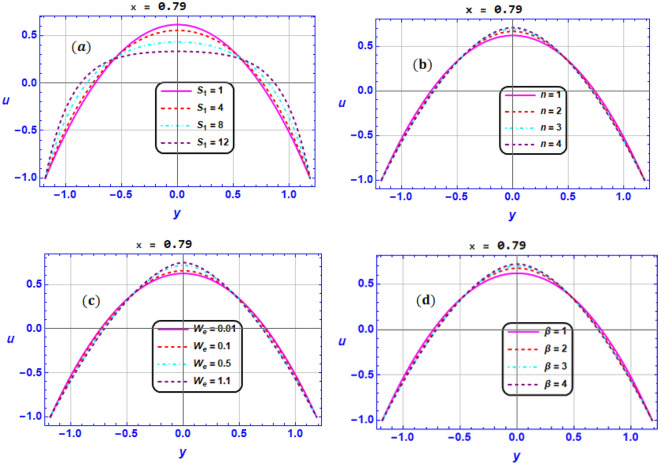
Variation of velocity profile $u$ for various values of (**a**) Strommer’s number ${S}_{1}$ (**b**) Power law index parameter $n$ (**c**) Weissenberg number $We$ (**d**) Viscosity parameter $\beta$ when ${G}_{rT}=0.5,{G}_{rC}=1,{G}_{rF}=0.8,{N}_{t}=0.3,{R}_{m}=0.5,E=0.5,{N}_{TC}=1.2,{N}_{CT}=0.9,F=0.2,{R}_{e}=0.5,{\gamma }_{1}=0.2,{\gamma }_{2}=0.3,{\gamma }_{3}=0.4, {\beta }_{1}=1,{\beta }_{2}=2,{\beta }_{3}=3, We=0.3.$

**Figure 3:**
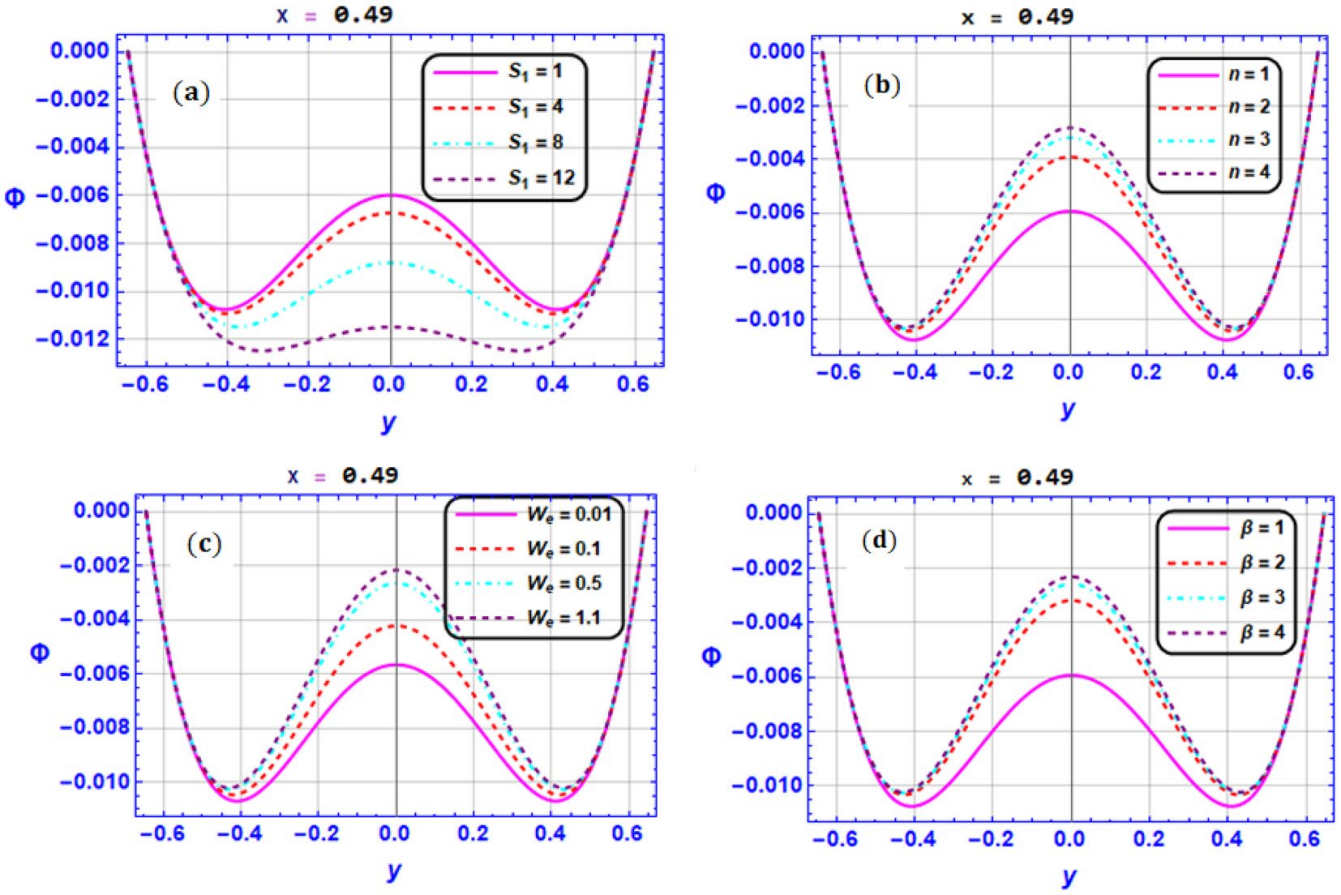
Variation of magnetic field profile $\Phi$ for various values of (**a**) Strommer’s number ${S}_{1}$ (**b**) Power law index parameter $n$ (**c**) Weissenberg number $We$ (**d**) Viscosity parameter $\beta$ when ${G}_{rT}=0.5,{G}_{rC}=1,{G}_{rF}=0.8,{N}_{t}=0.3,{R}_{m}=0.5,E=0.5,{N}_{TC}=1.2,{N}_{CT}=0.9,F=0.2,{R}_{e}=0.5,{\gamma }_{1}=0.2,{\gamma }_{2}=0.3,{\gamma }_{3}=0.4, {\beta }_{1}=1,{\beta }_{2}=2,{\beta }_{3}=3, We=0.3.$

**Figure 4:**
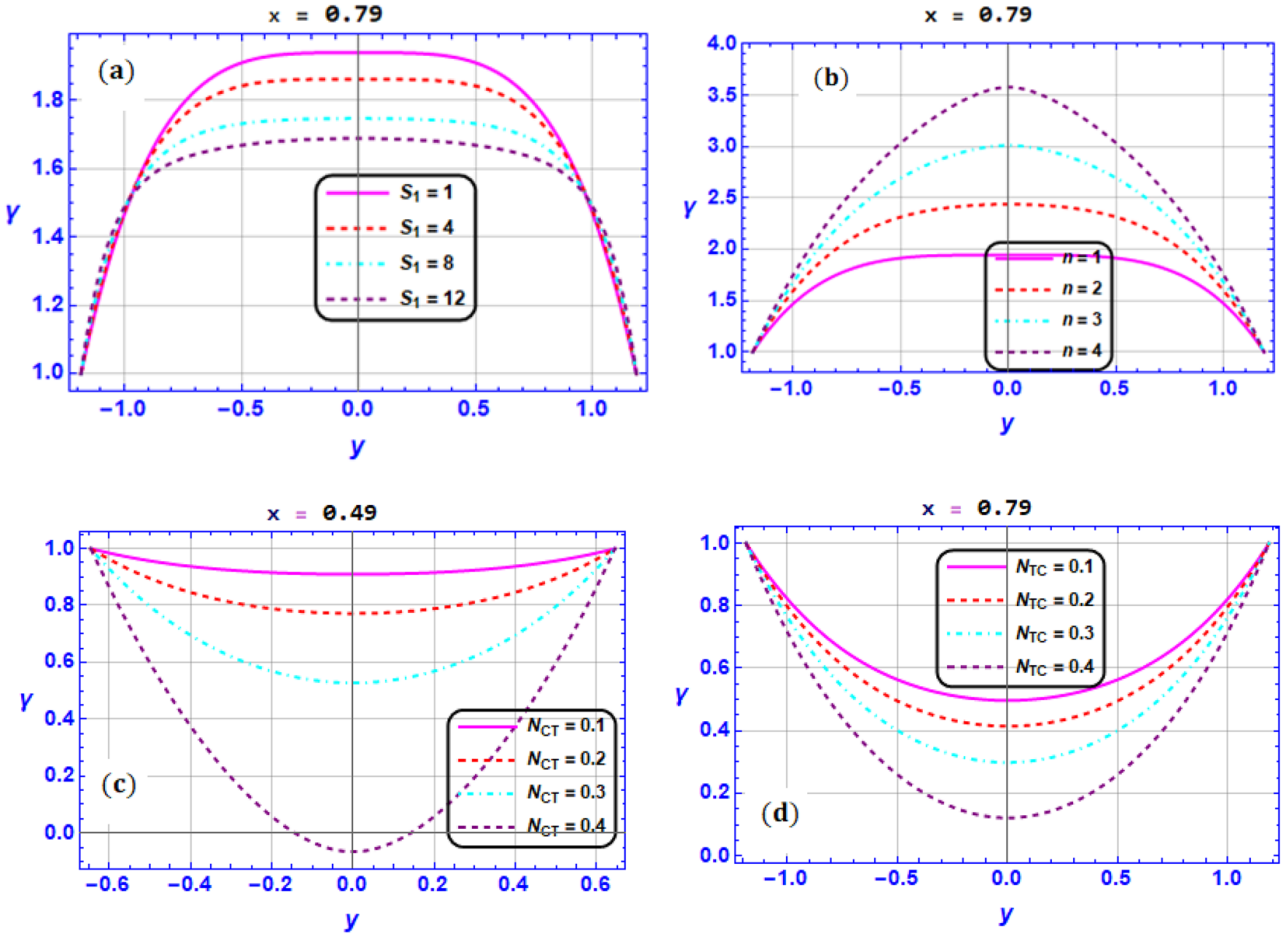
Variation of concentration profile $\gamma$ for various values of (**a**) Strommer’s number ${S}_{1}$ (**b**) Power law index parameter $n$ (**c**) Soret number ${N}_{CT}$ (**d**) Dufour number ${N}_{TC}$ when ${G}_{rT}=0.5,{G}_{rC}=1,{G}_{rF}=0.8,{N}_{t}=0.3,{R}_{m}=0.5,E=0.5,{N}_{TC}=1.2,{N}_{CT}=0.9,F=0.2,{R}_{e}=0.5,{\gamma }_{1}=0.2,{\gamma }_{2}=0.3,{\gamma }_{3}=0.4, {\beta }_{1}=1,{\beta }_{2}=2,{\beta }_{3}=3, We=0.3.$

**Figure 5:**
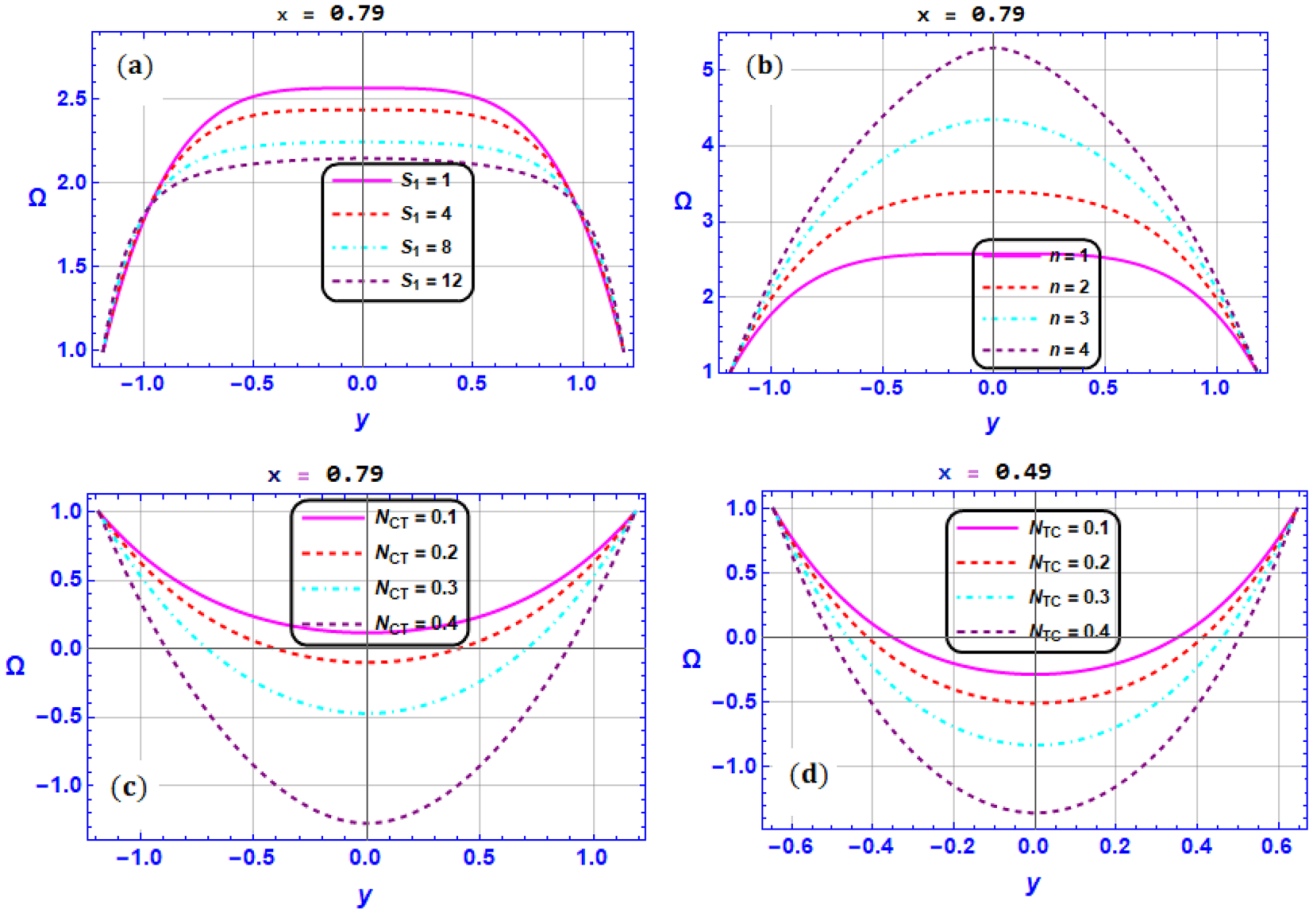
Variation of nanoparticles fraction profile $\Omega$ for various values of (**a**) Strommer’s number ${S}_{1}$ (**b**) Power law index parameter $n$ (**c**) Soret number ${N}_{CT}$ (**d**) Dufour number ${N}_{TC}$ when ${G}_{rT}=0.5,{G}_{rC}=1,{G}_{rF}=0.8,{N}_{t}=0.3,{R}_{m}=0.5,E=0.5,{N}_{TC}=1.2,{N}_{CT}=0.9,F=0.2,{R}_{e}=0.5,{\gamma }_{1}=0.2,{\gamma }_{2}=0.3,{\gamma }_{3}=0.4, {\beta }_{1}=1,{\beta }_{2}=2,{\beta }_{3}=3, We=0.3.$

**Figure 6:**
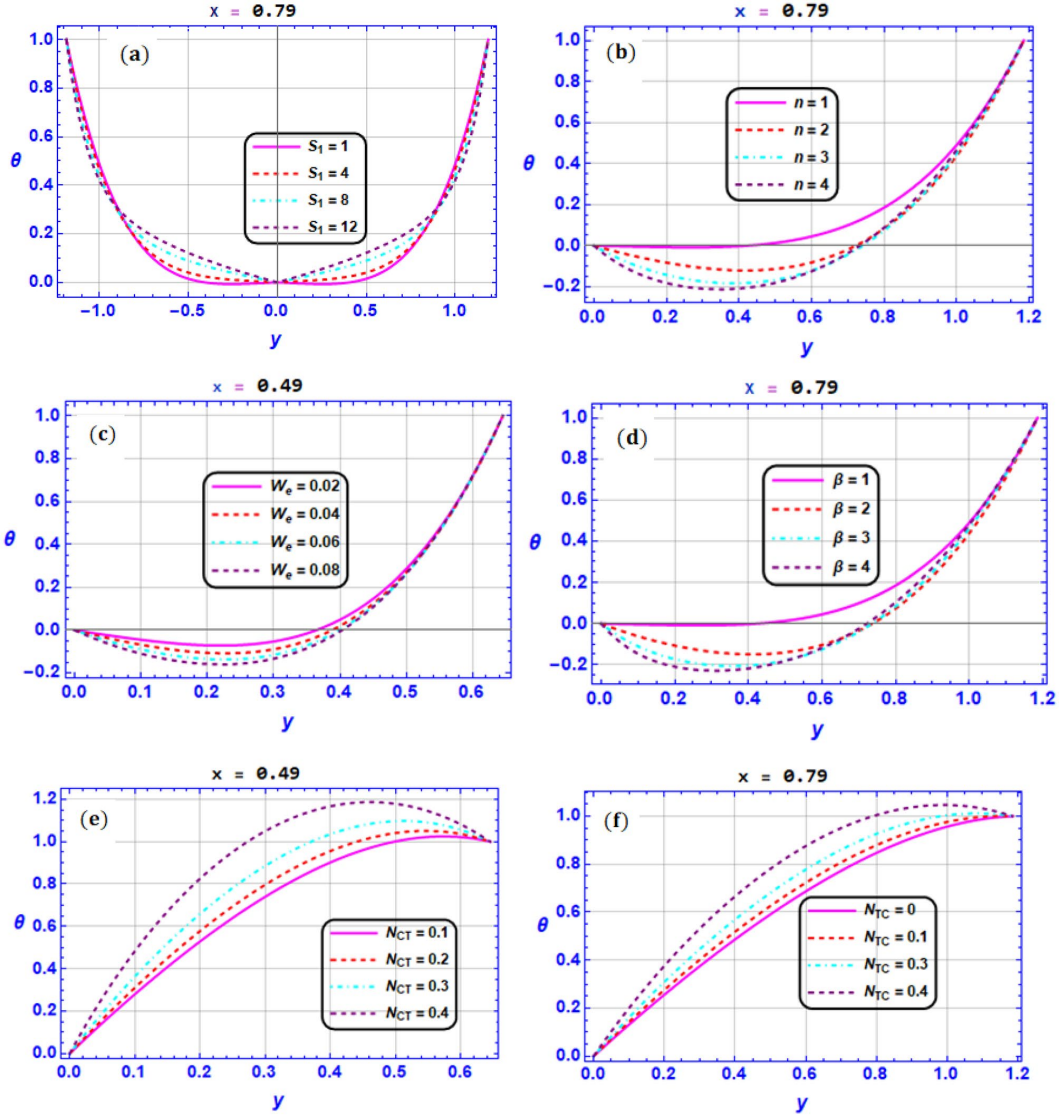
Variation of temperature profile $\theta$ for various values of (**a**) Strommer’s number ${S}_{1}$ (**b**) Power law index parameter $n$ (**c**) Weissenberg number $We$ (**d**) Viscosity parameter $\beta$ (**e**) Soret number ${N}_{CT}$ (**f**) Dufour number ${N}_{TC}$ when ${G}_{rT}=0.5,{G}_{rC}=1,{G}_{rF}=0.8,{N}_{t}=0.3,{R}_{m}=0.5,E=0.5,{N}_{TC}=1.2,{N}_{CT}=0.9,F=0.2,{R}_{e}=0.5,{\gamma }_{1}=0.2,{\gamma }_{2}=0.3,{\gamma }_{3}=0.4, {\beta }_{1}=1,{\beta }_{2}=2,{\beta }_{3}=3, We=0.3.$

**Figure 7:**
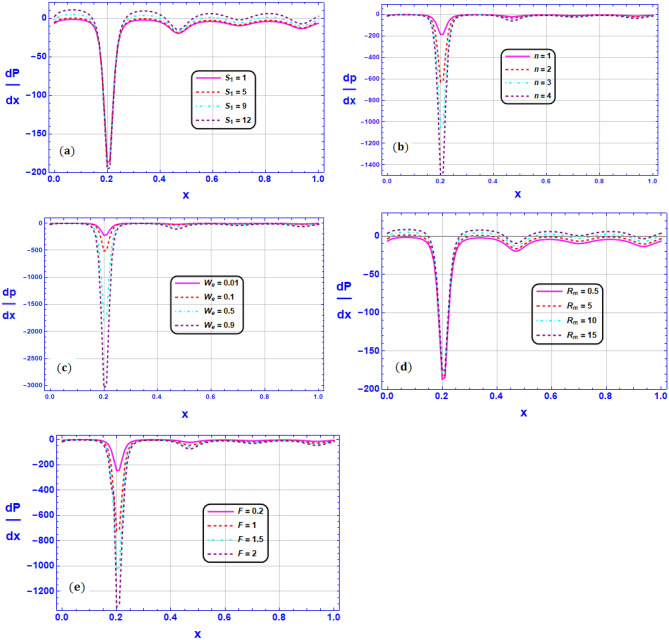
Variation of pressure gradient $\frac{dp}{dx}$ for various values of (**a**) Strommer’s number ${S}_{1}$ (**b**) Power law index parameter $n$ (**c**) Weissenberg number $We$ (**d**) Magnetic Reynolds number ${R}_{m}$ (**e**) Cross section parameter $F$ when ${G}_{rT}=0.5,{G}_{rC}=1,{G}_{rF}=0.8,{N}_{t}=0.3,{R}_{m}=0.5,E=0.5,{N}_{TC}=1.2,{N}_{CT}=0.9,F=0.2,{R}_{e}=0.5,{\gamma }_{1}=0.2,{\gamma }_{2}=0.3,{\gamma }_{3}=0.4, {\beta }_{1}=1,{\beta }_{2}=2,{\beta }_{3}=3, We=0.3.$

**Figure 8:**
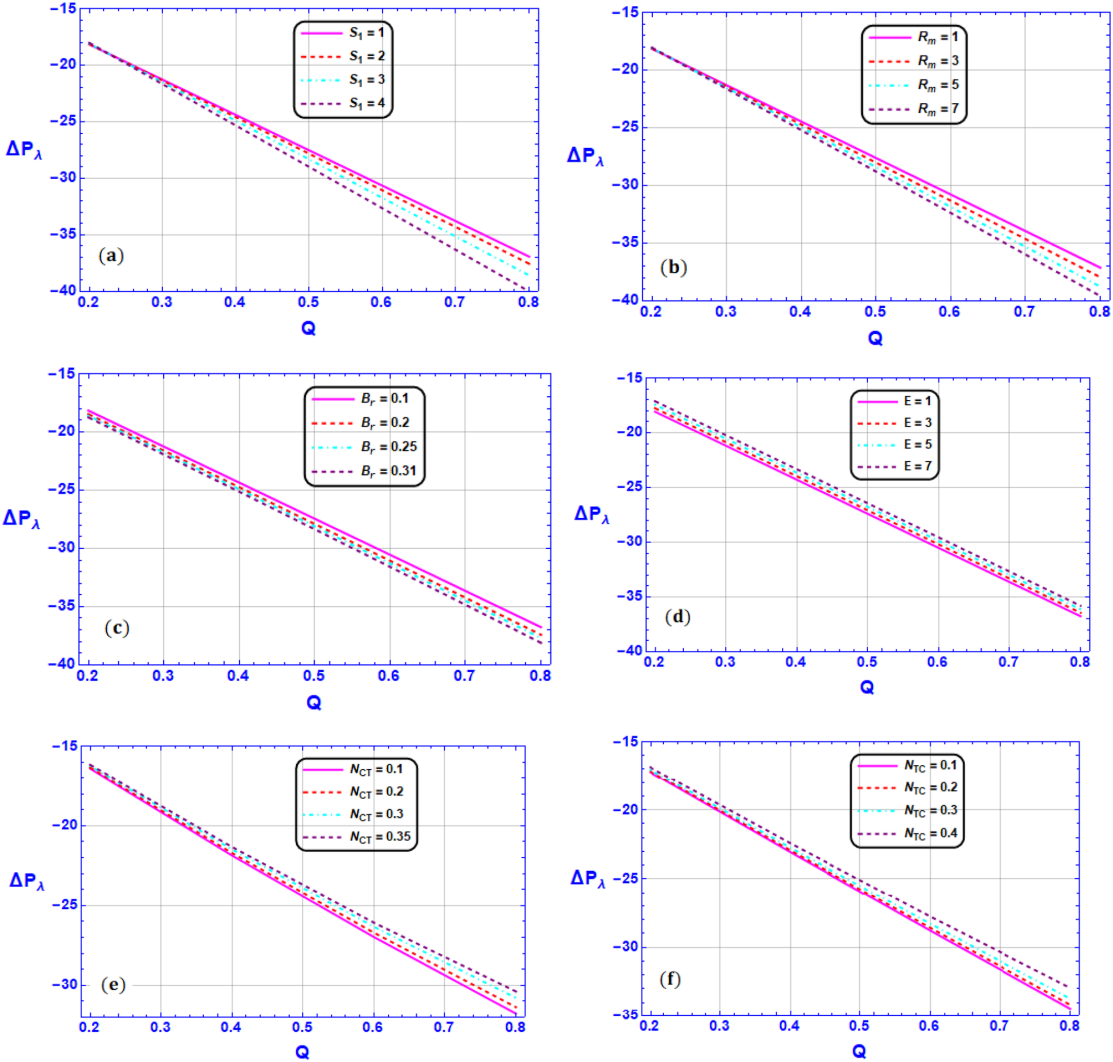
Variation of pressure rise $\Delta {P}_{\lambda }$ for various values of (**a**) Strommer’s number ${S}_{1}$ (**b**) Magnetic Reynolds number ${R}_{m}$ (**c**) Brinkman number $Br$ (**d**) Electric field parameter $E$ (**e**) Soret number ${N}_{CT}$ (**f**) Dufour number ${N}_{TC}$ when ${G}_{rT}=0.5,{G}_{rC}=1,{G}_{rF}=0.8,{N}_{t}=0.3,{R}_{m}=0.5,E=0.5,{N}_{TC}=1.2,{N}_{CT}=0.9,F=0.2,{R}_{e}=0.5,{\gamma }_{1}=0.2,{\gamma }_{2}=0.3,{\gamma }_{3}=0.4, {\beta }_{1}=1,{\beta }_{2}=2,{\beta }_{3}=3, We=0.3.$

**Figure 9:**
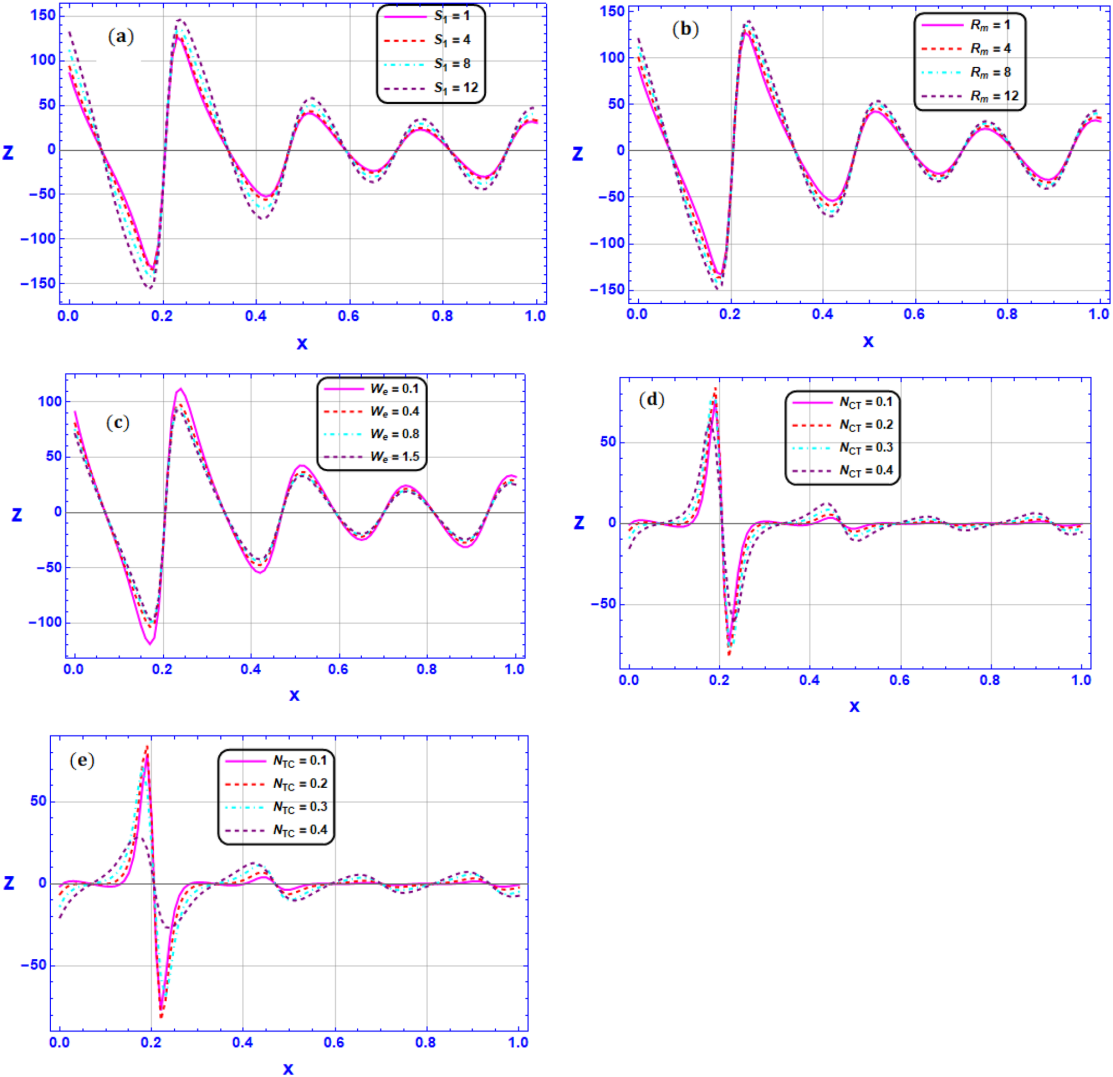
Variation of heat transfer rate $Z$ for various values of (**a**) Strommer’s number ${S}_{1}$ (**b**) Magnetic Reynolds number ${R}_{m}$ (**c**) Weissenberg number $We$ (**d**) Soret number ${N}_{CT}$ (**e**) Dufour number ${N}_{TC}$ when ${G}_{rT}=0.5,{G}_{rC}=1,{G}_{rF}=0.8,{N}_{t}=0.3,{R}_{m}=0.5,E=0.5,{N}_{TC}=1.2,{N}_{CT}=0.9,F=0.2,{R}_{e}=0.5,{\gamma }_{1}=0.2,{\gamma }_{2}=0.3,{\gamma }_{3}=0.4, {\beta }_{1}=1,{\beta }_{2}=2,{\beta }_{3}=3, We=0.3.$

**Figure 10:**
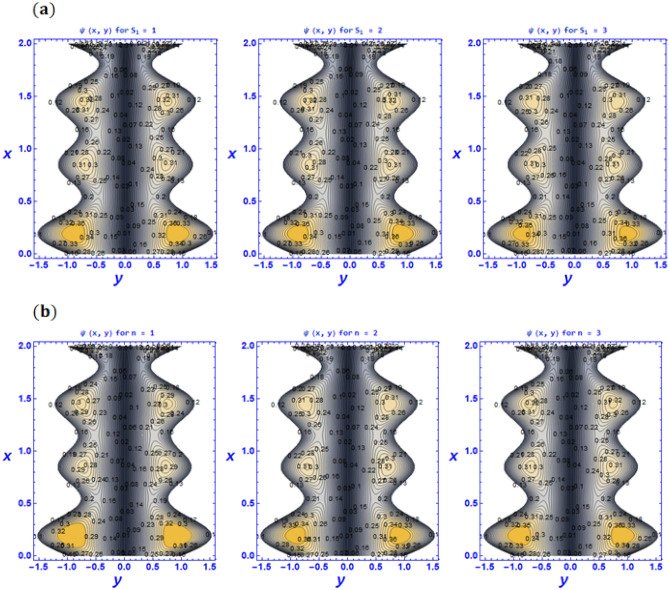

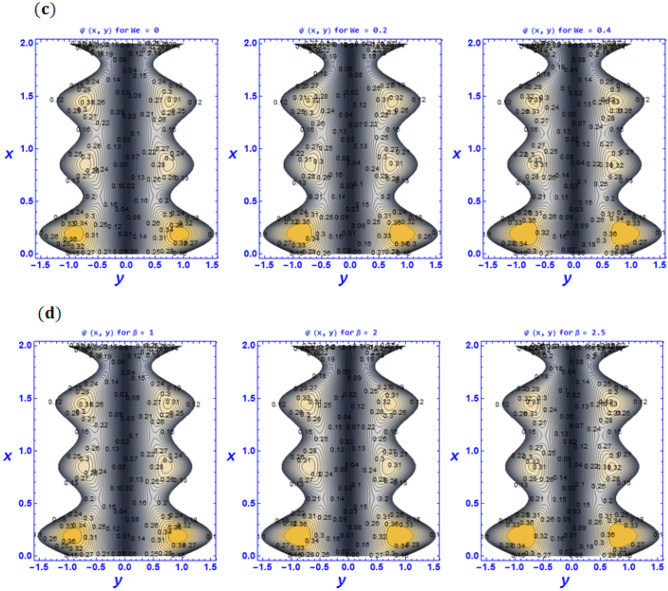
Variation of pattern phenomena $\psi \left(x,y\right)$ for various values of (**a**) Strommer’s number ${S}_{1}$ (**b**) Power law index parameter $n$ (**c**) Weissenberg number $We$ (**d**) Viscosity parameter $\beta$ when ${G}_{rT}=0.5,{G}_{rC}=1,{G}_{rF}=0.8,{N}_{t}=0.3,{R}_{m}=0.5,E=0.5,{N}_{TC}=1.2,{N}_{CT}=0.9,F=0.2,{R}_{e}=0.5,{\gamma }_{1}=0.2,{\gamma }_{2}=0.3,{\gamma }_{3}=0.4, {\beta }_{1}=1,{\beta }_{2}=2,{\beta }_{3}=3, We=0.3.$

**Figure 11:**
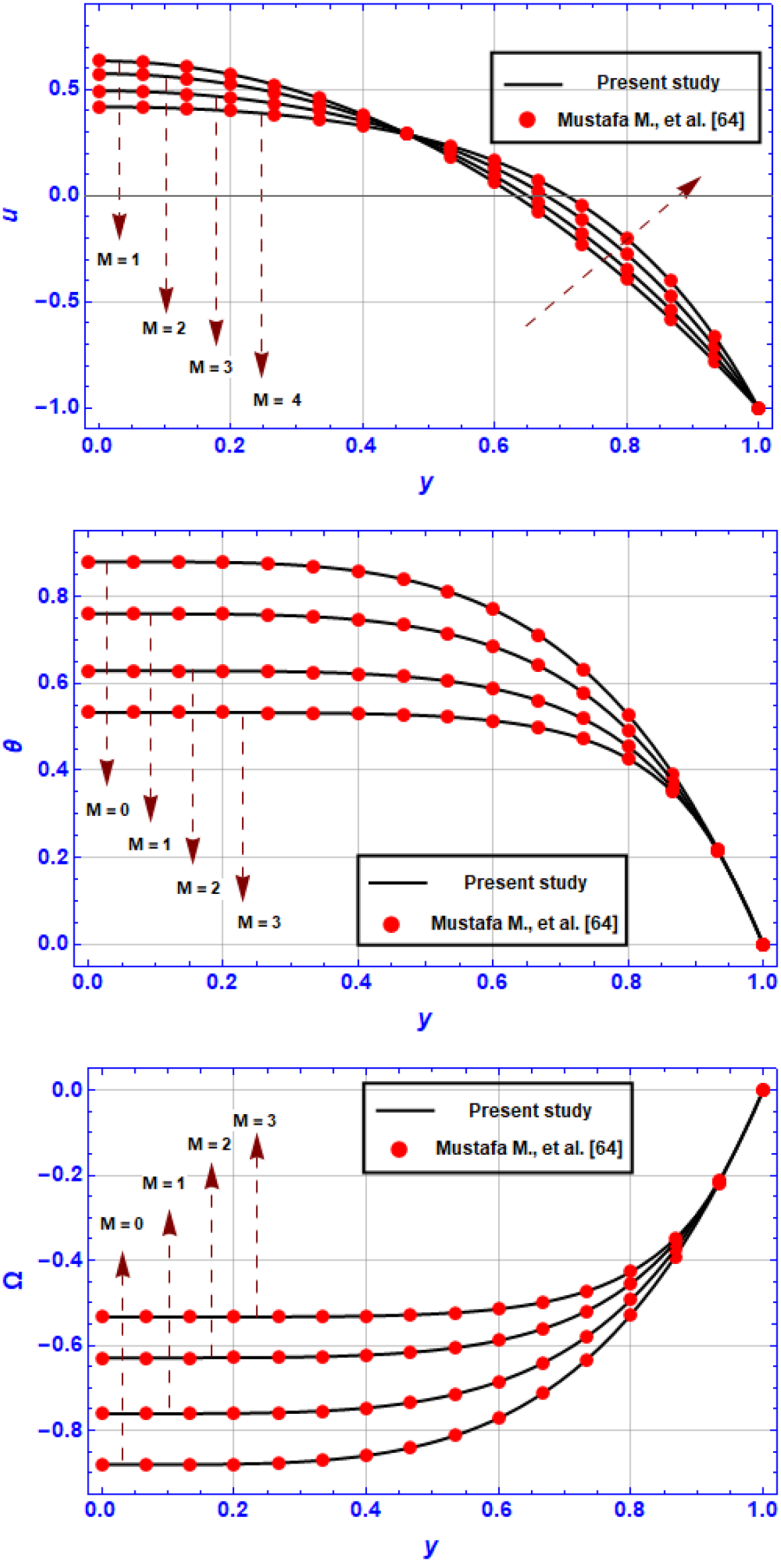
Comparison outcomes with the earlier published work by Mustafa M. et al.^64^.

A mathematical model has been created to assess the combined impacts of DD convection and an induced magnetic field on the peristaltic pumping of BN-EG nanofluid flow in a two-dimensional micro-channel with propagation of complicated waves. We organized and explained our obtained outcomes for different values of the flow parameters included in the investigated problem using graphical representations. But one of the crucial tasks is to validate the outcomes of our developed code with those of earlier studies that have been published. For this purpose of validation, we have contrasted the results with those that had previously reported by Mustafa M. et. al.^64^. we made an effort to validate the findings of axial velocity, temperature and concentration profile behavior under the effect of magnetic field coefficient $M$ for the uniform channel case and when $We=0,{\beta }_{1}={2, \gamma }_{1}=0.2,{{\gamma }_{2}=0,{\gamma }_{3}=0,G}_{rC}=0$ in the absence of the solute concentration equation. This validation is presented in Fig. 11. Based on these figures, we could conclude that our findings are in good consistent with previous study by Mustafa M. et al.^64^. This finding provides adequate validation for this analysis. In this section, we investigated the effect of key electro-magnetic and hydrodynamic parameters on the flow variables in Figs. 2, 3, 4, 5, 6, 7, 8, 9 and 10 by visualizing numerical results that analyzed by utilizing Mathematica symbolical software. It's important to note that the typical parameter values for all obtained figures are ${G}_{rT}=0.5,{G}_{rC}=1,{G}_{rF}=0.8,{N}_{t}=0.3,{R}_{m}=0.5,E=0.5,{Br=0.1,N}_{TC}=1.2,{N}_{CT}=0.9,F=0.2,{R}_{e}=0.5,{\gamma }_{1}=0.2,{\gamma }_{2}=0.3,{\gamma }_{3}=0.4, {\beta }_{1}=1,{\beta }_{2}=2,{\beta }_{3}=3, We=0.3.$ Fig. 2a–d illustrates the impact of respectively (a) Strommer’s number ${S}_{1}$ (b) Power law index parameter $n$ (c) Weissenberg number $We$ (d) Viscosity parameter $\beta$ on axial velocity $u$. These illustrations are conducted at the section $x=0.79$. In Fig. 2a, it is shown that for higher values of ${S}_{1}$, velocity diminishes near the center of the channel $\left(y\in \left[-\mathrm{0.5,0.5}\right]\right)$, but a different behavior is shown near the walls. Furthermore, it is observed that the velocity tends to enhance for greater values of $n$ in the domain $y\in \left[-\mathrm{0.5,0.5}\right]$ and the opposite manner is shown in the region $y\in \left[-1,-0.5\right]\bigcup \left[\mathrm{0.5,1}\right]$ (see Fig. 2b). In the same context, it is portrayed from Fig. 2c and Fig. 2d that the magnitude value of the velocity profile augmented due to maximizing behavior of $We$ and $\beta$ when $y\in \left[-\mathrm{0.5,0.5}\right]$, but the reverse behavior is spotted when $y\in \left[-1,-0.5\right]\bigcup \left[\mathrm{0.5,1}\right]$. *Physically*, Weissenberg number is the proportion of the fluid's relaxation time to a particular process time, consequently, raising the Weissenberg number will result in a longer relaxation period, which will allow for easier flow and an increase in the velocity field.

Figure 3a–d depicts the effect of pertinent parameters on the magnetic force function. These illustrations are conducted at the section $x=0.49$. Physical impact of ${S}_{1}$ on the magnetic field function is dedicated in Fig. 3a. It is found in this figure that the magnetic function tends to reduce in the region $y\in \left[-\mathrm{0.4,0.4}\right]$ and weak behavior is noted near the walls. It is witnessed in Fig. 3b–d that as $n,We$ and $\beta$ values boost, the magnetic force function's magnitude tends to significantly rise in the region $y\in \left[-\mathrm{0.4,0.4}\right]$ but the behavior near the walls tends to decay.

Figure 4a–d present the variation in concentration profile for various parameters, respectively (a) Strommer’s number ${S}_{1}$ (b) Power law index parameter $n$ (c) Soret number ${N}_{CT}$ (d) Dufour number ${N}_{TC}$. These representations are displayed at different two sections $x=\mathrm{0.49,0.79}$. To characterize the behavior of concentration profile for various values of the Strommer’s number ${S}_{1}$ and power law index parameter $n$ Fig. 4a and b are presented. It can be viewed that the concentration profile tends to reduce as ${S}_{1}$ is altered. On the contrary the behavior of concentration revealed that the maximizing of power law index parameter $n$ causes an augmentation in the concentration distribution. As shown in Fig. 4c and d, the concentration profile tends to diminish as the values of the ${N}_{CT}$ and ${N}_{TC}$ parameters enhance. *Physically*, this is due to the interaction between spontaneous motion and the solid nanoparticles' random collision and micro-mixing behaviors, which lowering the solute concentration and disperses the solid nanoparticles.

Figure 5a–d display the variation in the nanoparticle fraction versus ${S}_{1},n,{N}_{CT}{,N}_{TC}$.This impact is examined at two different section, namely $x=0.\mathrm{49,0.79}$. Here, the fraction of nanoparticles get lower features as ${S}_{1}$ is maximized (see Fig. 5a). While the nanoparticles’ reaction are maximized as $n$ is growing as witnessed in Fig. 5b. As shown in Fig. 5c and d, the nanoparticles fraction tends to reduce as the values of the ${N}_{CT}$ and ${N}_{TC}$ parameters enhance. *Physically*, this is due to the interaction between spontaneous motion and the solid nanoparticles’ random collision and micro-mixing behaviors, which lowering the solute concentration and disperses the solid nanoparticles.

Figure 6a–f discloses the impacts of ${S}_{1},n,We,\beta ,{N}_{CT}{,N}_{TC}$ at $x=0.49, 0.79$ on the temperature distribution. It is illustrated from Fig. 6a that the growing in ${S}_{1}$ causes an enhancement in temperature in the interval $y\in \left[-\mathrm{0.9,0.9}\right]$ but the reverse situation is sustained in the remaining part of the microchannel. Additionally, the increase in $n,We,\beta$ results in a reduction in the temperature profile as depicted in Fig. 6b–d. Also, it is found in Fig. 6e–f that increases in the Soret and Dufour number result in an augmentation in the temperature profile. This is due to the fact that the temperature is directly related to the restrictions and limitations of the Soret and Dufour number. We emphasize that numerous other analytical studies, such as those by^62^, have detected this behavior.

Figure 7a–e display the variation of pressure gradient $\frac{dP}{dx}$ for various values of respectively, (a) Strommer’s number ${S}_{1}$ (b) Power law index parameter $n$ (c) Weissenberg number $We$ (d) Magnetic Reynolds number ${R}_{m}$ (e) Cross section parameter $F$ when ${G}_{rT}=0.5,{G}_{rC}=1,{G}_{rF}=0.8,{N}_{t}=0.3,{R}_{m}=0.5,E=0.5,{N}_{TC}=1.2,{N}_{CT}=0.9,F=0.2,{R}_{e}=0.5,{\gamma }_{1}=0.2,{\gamma }_{2}=0.3,{\gamma }_{3}=0.4, {\beta }_{1}=1,{\beta }_{2}=2,{\beta }_{3}=3, We=0.3$ portrayed along the axial coordinates $(x-axis)$. With noticeably higher amplitudes computed at the entry zone to the micro-channel $\left(x\approx 0.2\right)$, the sophisticated biomimetic propulsion can be predicted to have an undulating form for pressure gradient diagrams. The physical effects of ${S}_{1},{R}_{m}$ on $\frac{dP}{dx}$ are displayed in Fig. 7a and d. It is observed from these figures that $\frac{dP}{dx}$ is augmented as ${S}_{1},{R}_{m}$ is enhanced. On the contrary, the velocity gradients are increasing as $n,We$ and $\beta$ are altered which causes an augmentation in the fluid friction and as a result $\frac{dP}{dx}$ is diminishing.

Figure 8a–e present the variation of pressure rise $\Delta {P}_{\lambda }$ for various values of (a) Strommer’s number ${S}_{1}$ (b) Magnetic Reynolds number ${R}_{m}$ (c) Brinkman number $Br$ (d) Electric field parameter $E$ (e) Soret number ${N}_{CT}$ (f) Dufour number ${N}_{TC}$ and sketched with the averaged volumetric flow parameter $Q$ when ${G}_{rT}=0.5,{G}_{rC}=1,{G}_{rF}=0.8,{N}_{t}=0.3,{R}_{m}=0.5,E=0.5,{N}_{TC}=1.2,{N}_{CT}=0.9,F=0.2,{R}_{e}=0.5,{\gamma }_{1}=0.2,{\gamma }_{2}=0.3,{\gamma }_{3}=0.4, {\beta }_{1}=1,{\beta }_{2}=2,{\beta }_{3}=3, We=0.3.$ The human body has a significant mechanism called peristaltic pumping that facilitates in the circulation of several biological fluids. Further, microvascular vasomotion, the movement of urine from the kidney to the bladder, cilia mobility, and a variety of other motions are examples of this type of motion. It was divided into four parts so that it could be observed better vigorously, namely pumping by peristaltic motion $(\Delta {P}_{\lambda } > 0,Q > 0)$, regressive pumping region $(\Delta {P}_{\lambda } > 0,Q < 0)$, free pumping zone $(\Delta {P}_{\lambda } < 0,Q < 0$ and the region of co-pumping $\left(\Delta {P}_{\lambda } < 0,Q > 0\right)$. It is noticed from Fig. 8a–c that an augmentation in Strommer’s number ${S}_{1}$, Magnetic Reynolds number ${R}_{m}$ and Brinkman number $Br$ substantially causes a reduction in the pressure rise in the region of co-pumping $(\Delta {P}_{\lambda } < 0,Q > 0)$. While, in the peristaltic co-pumping region $\left(\Delta {P}_{\lambda } < 0,Q > 0\right)$, the opposite pattern is prophesied for higher values of Electric field parameter $E$, Soret number ${N}_{CT}$ and Dufour number ${N}_{TC}$ as shown in Fig. 8d and e.

Figure 9a–e Illustrate the evolution of heat transfer rate $Z$ along the channel length for respective variations in Strommer’s number ${S}_{1}$, Magnetic Reynolds number ${R}_{m}$, Weissenberg number $We$, Soret number ${N}_{CT}$ and Dufour number ${N}_{TC}$ respectively at ${G}_{rT}=0.5,{G}_{rC}=1,{G}_{rF}=0.8,{N}_{t}=0.3,{R}_{m}=0.5,E=0.5,{N}_{TC}=1.2,{N}_{CT}=0.9,F=0.2,{R}_{e}=0.5,{\gamma }_{1}=0.2,{\gamma }_{2}=0.3,{\gamma }_{3}=0.4, {\beta }_{1}=1,{\beta }_{2}=2,{\beta }_{3}=3, We=0.3.$ The outcomes revealed that the maximizing in the ${S}_{1},{R}_{m}$ causes a reduction the heat transfer distribution while the heat transfer rates are maximized as $We$ is growing. In the same context, Fig. 9d and e discloses the impacts of ${N}_{CT}$ and ${N}_{TC}$ on heat transfer rate $Z$. Heat transfer rate is enhanced throughout the micro-channel length by raising the Dufour or (diffusion-thermo impact) and the Soret or (thermal diffusion impact) as illustrated in Fig. 9d and e. Also, it is worth noticeable that greatly enhancement in heat transfer rate is observed in the region $x=0.2$. *Physically*, an energy flux can be created by thermal gradients as well as composition gradients when heat and mass transfer happen at the same time in a flowing fluid. The Dufour or diffusion-thermo impact is the name given to the energy flux brought on by a composition gradient. On the contrary, the Soret or heat diffusion impact is embodied by the fact that mass fluxes can also be produced by thermal gradients.

Trapping is another essential characteristic of rheological behavior in the peristaltic transport of fluid. Under certain situations, the configuration of a propagated bolus appears in the form of streamlines near to the boundary walls in this phenomenon. They move in the direction of waves that have the same speed as peristaltic waves and completely rely on the characteristics of peristaltic waves that are existent at the boundary walls. It is capable of determining reflux properties as well as vorticity growth and blood circulation density in peristaltic flows. Moreover, Fig. 10a–d have been used to discuss the trapping phenomena. To depict the streamlines for various values of the Strommer’s number and power law index parameter $n$ Fig. 10a and b are presented. It can be seen that the size of the trapping bolus tends to decrease while the density of trapping bolus increases with an enhancement in the power law index and Strommer’s number. Streamlines for different values of the Weissenberg number $We$ are prepared in Fig. 10c. It is observed that Weissenberg number $We$ which is the proportion of the fluid's relaxation time to a particular process time; consequently, raising the Weissenberg number will result in a longer relaxation period, which will allow for easier blood flow. Therefore, when we boost the Weissenberg number, the size of the trapping bolus grows as well. According to Akbar and Nadeem^63^, a similar effect has been established. In addition, similar behavior is witnessed for larger values of viscosity parameter $\beta$ (see Fig. 10d).

## Conclusions

A mathematical model has been constructed for peristaltic electrically conducting of BN-EG nanofluid flow in a micro-channel with propagation of complicated waves enforced at the walls, which was inspired by recent advancements in ocular physiological delivery systems. So far, little attention has been paid to the features of BN nanoparticles suspended in EG mixture as a base fluid. This paper will contribute to filling that void by providing a comprehensive analysis of the peristaltic transport of BN-EG nanofluid. The key conclusions that can be drawn are as follows:

- The axial flow is powerfully accelerated, and the magnetic field function is significantly increased as the Power law index parameter $n$ and Weissenberg number $We$ augment, while the temperature profile and pressure gradient minimize. It is also worth noting that increasing the Power law index parameter $n$ and the Weissenberg number $We$ causes the boluses near the upper and lower channel walls tends to vanish but the contours of the streamlines is enhanced as $We$ is altered.
- As Strommer’s number ${S}_{1}$ rises, axial velocity diminishes in the region $y\in \left[-\mathrm{0.5,0.5}\right]$, the pressure gradient increases noticeably, the pressure rise in the co-pumping region is greatly reduced, and many boluses (trapped regions) are produced in the channel's central zone.
- With increasing viscosity parameter, axial velocity is maximized, and magnetic field function is augmented, while for higher values of viscosity parameter the temperature distribution is minimized. Further, with greater viscosity parameter values, streamline patterns are more flexible, which diminishes bolus numbers in the channel's central core.
- The temperature profile is found to rise when the Soret and Dufour numbers rise, whereas the opposite effect is apparent for the concentration of solute and nanoparticle volume fraction.
- The current study’s mathematical analysis and rheological characteristics provide a durable base for more wide range of biological models utilizing by sophisticated peristaltic pumps. Further to that, the induced magnetic field and DD convection have a major impact on peristaltic pump performance.
- According to Fig. 11 at $We=0,{\beta }_{1}={2, \gamma }_{1}=0.2,{\gamma }_{2}=0,{\gamma }_{3}=0,{G}_{rC}=0$ in the absence of the solute concentration equation., premium agreement are noted between the results from our study and those that have already been published in Ref.^64^.

The current findings are pertinent to clarifying the fluid dynamics of innovative medication delivery systems that have been suggested. However, the current study considered the effects of induced magnetic field and DD- convection while in the future it can be generalized by assuming the cases of the variable magnetic field and non-linear radiation flux as well as slip effects at the microchannel walls. Additionally, entropy generation minimization is crucial for pharmacology improving nano-electromagnetohydrodynamic pumping designs. Therefore, all of these topics will be covered in the future.

## Data Availability

The datasets generated and/or analyzed during the current study are not publicly available due [All the required data are only with the corresponding author] but are available from the corresponding author on reasonable request.

## List of symbols

$\left(\mathcal{U},V\right)$: 2-Dimensional flow’s axial velocity
$$t$$: Time
$$\lambda$$: The wavelength
$$\widehat{a}$$: Wall amplitude
$T$: The fluid temperature
$c$: The speed of the wave
$$C$$: The concentration
${D}_{T}$: The coefficient of thermophoretic diffusion
${\beta }_{C}$: The coefficient of volumetric solute expansion
${D}_{B}$: The coefficient of Brownian diffusion
${D}_{s}$: The diffusively of solute
${A}_{1}$: The “first Rivlin-Erickson tensor”
${\left(\rho c\right)}_{p}$: The effective nanoparticle heat capacity
${\rho }_{f0}$: The fluid density
$\Theta$: The volume fraction of nanoparticle
$\eta$: The perceived viscosity
${\eta }_{0}{,\eta }_{\infty }$: The viscosities with zero and infinite shear rates
$grad V$: The gradient of velocity
$\alpha$: The fluid material parameter
$\left(\overline{H },-\overline{H }\right)$: The upper and lower walls
$$\beta$$: Viscosity parameter’s
$$Nb$$: Brownian motion
$$Pr$$: Prandtl number
$$Le$$: Lewis number
$Nt$: The parameter thermophoresis motion
$Ln$: The Lewis number of nanoparticle
$\delta$: The numbers of the wave
${R}_{m}$: Magnetic Reynolds number
$$Re$$: Reynolds number
${\mu }_{e}$: Permeability to magnetic fields
$\chi$: The magnetic diffusion
g: The acceleration of the gravity
$\sigma$: The electrical conductivity
${\varvec{E}}$: The induction of an electric field
${\varvec{J}}$: The density of the current
${\varvec{V}}$: The vector of the velocity
${\beta }_{T}$: The coefficient of volumetric heat expansion
${\widehat{b}}_{0}$: The half of the conduit’s width at an axial distance
$${D}_{TC}$$: Dufour diffusion
$${D}_{CT}$$: Soret diffusively
$${\widehat{b}}_{1}$$: Constant
${\left(\rho c\right)}_{f}$: Fluid heat capacity
${\rho }_{p}$: The density of nanoparticles
${\rho }_{f}$: Symbolizes fluid density
$T$: The temperature of the fluid
$n$: Index of the non-dimensional power law
$\left({\overline{\gamma }}_{1},{\overline{\gamma }}_{2},{\overline{\gamma }}_{3}\right)$: The complex wave amplitudes
$\dot{\gamma }$: The shear rate
${G}_{rF}$: Nanoparticle Grashof number
${G}_{rT}$: Thermal expansion Grashof number
${G}_{rc}$: The Grashof number of solute
$$\theta$$: Dimensionless temperature
$\Omega$: Volume fraction distribution
$\gamma$: The concentration of solute
$${N}_{TC}$$: Dufour number
$${N}_{CT}$$: Soret number
$${S}_{1}$$: Strommer’s number
${H}_{0}$: The strength of magnetic field
